# Rigid Bronchoscopic Removal of Multiple Airway Foreign Bodies

**DOI:** 10.1155/DTE.4.95

**Published:** 1997

**Authors:** Charles F. Lano, Timothy L. Smith, Douglas K. Holmes

**Affiliations:** 1 S-2100 Medical Center North Vanderbilt University Medical Center Department of Otolaryngology Nashville TN 37232-2559 USA; 2 Wake County Hospital 3024 New Bern Ave. Ste. 305 Raleigh NC 27610 USA

## Abstract

Despite new equipment, such as the Hopkins rod-lens telescopes and optical forceps, foreign bodies in the airway continue to present a diagnostic and therapeutic challenge to the endoscopist. Airway foreign bodies are more common in children than adults and frequently, the patient may have aspirated more than one foreign body or the original foreign body fragments into pieces. Vegetable matter is the most frequently aspirated material by children. This material can swell as it absorbs water, it can cause an intense mucosal reaction and it can fragment during removal. A case with endoscopic photographs demonstrating these issues and a discussion are presented.


*Diagnostic and Therapeutic Endoscopy*, Vol. 4, pp. 95–99
Reprints available directly from the publisher
Photocopying permitted by license only

© 1997 OPA (Overseas Publishers Association)
Amsterdam B.V. Published under license
in The Netherlands under the Harwood Academic
Publishers imprint, part of The Gordon and
Breach Publishing Group.
Printed in Singapore.

### Rigid Bronchoscopic Removal of Multiple Airway Foreign Bodies

CHARLES F. LANO, Jr. [a], TIMOTHY L. SMITH [a,*] and DOUGLAS K. HOLMES [b]

[a] *S-2100 Medical Center North, Vanderbilt University Medical Center, Department of Otolaryngology, Nashville, TN 37232-2559, USA;* [b] *Wake County Hospital, 3024 New Bern Ave. Ste. 305, Raleigh, NC 27610, USA*

*(Received 17 February 1997; Revised 15 April 1997; In final form 6 May 1997)*

**Despite new equipment, such as the Hopkins rod-lens telescopes and optical forceps, foreign bodies in the airway continue to present a diagnostic and therapeutic challenge to the endoscopist. Airway foreign bodies are more common in children than adults and frequently, the patient may have aspirated more than one foreign body or the original foreign body fragments into pieces. Vegetable matter is the most frequently aspirated material by children. This material can swell as it absorbs water, it can cause an intense mucosal reaction and it can fragment during removal. A case with endoscopic photographs demonstrating these issues and a discussion are presented.**

*Keywords:* Airway, Bronchoscopy, Foreign body

### INTRODUCTION

Foreign bodies in the pediatric airway continue to present a diagnostic and therapeutic challenge to the endoscopist. In 1897 Gustav Killian performed the first endoscopic removal of a foreign body from the bronchus of a man using a 9 mm tube [1]. Chevalier Jackson, however, is credited with the development of the instruments and techniques which made rigid bronchoscopic removal of foreign bodies a successful procedure. By 1936 he reported that bronchoscopic removal of airway foreign bodies, which had previously been associated with a high mortality, was successful in 98% of cases [2,3]. The development of the Hopkins rod-lens telescope in the 1970s was the next major improvement for the rigid bronchoscopic removal of foreign bodies. It provided improved illumination and visualization. Telescopic guided forceps have further improved these procedures and have resulted in lower complication rates [4].

Foreign body aspiration is a life threatening condition which more commonly affects children than adults. Most commonly these are children under 4 years of age. Boys are affected more often than girls by a ratio of 2:1. There are approximately 3000 fatal cases in the United States each

* Corresponding author. Tel.: (615) 322-7267. Fax: (615) 343-7604.

year [5]. Early recognition of this potentially fatal situation is imperative to prevent complications. Appropriate emergency management of the child with complete airway obstruction is somewhat controversial, but current recommendations include back blows for children under 1 year of age and the Heimlich maneuver for all others [6].

Initially the aspirated foreign body may cause violent coughing paroxysms, wheezing, choking and gagging. These initial symptoms may be followed by a period of time when the child is relatively asymptomatic. At this point, the child may present to the physician with minimal distress [7]. If untreated, patients may develop cough, hemoptysis, pneumonia, lung abscess, fever and malaise. McGuirt *et al.* found that only 61% of children with tracheobronchial foreign bodies had abnormal physical findings, most commonly decreased breath sounds or wheezing [8].

Vegetable matter is uniformly the most common foreign body found in the pediatric airway. Peanuts and other nuts account for a large percentage of these cases. There is a wide array of other foreign bodies aspirated including pins, small plastic objects, stones, bones and glass. Most foreign bodies of the airway pass the larynx and trachea to become lodged in the main bronchi or segmental bronchi. In a historical review of the experience at Johns Hopkins, laryngeal foreign bodies comprised 1.5% of airway foreign bodies, tracheal foreign bodies accounted for 12% and the remainder were in the bronchi [9].

Neck and posteroanterior and lateral chest radiographs are the most helpful studies in patients with a suspected airway foreign body. Even though false negative chest radiographs may be seen in up to 40%, air trapping (obstructive emphysema) on the side of the foreign body, atelectasis and pneumonia are frequently noted [7].

For the removal of most airway foreign bodies it is preferable to allow the patient to breath spontaneously under general anesthesia. This provides a controlled setting and the patient is able to maintain adequate oxygenation using their own respirations. Induction of anesthesia is performed using a mask. Preoperative sedation is avoided. Rarely, the airway is severely obstructed by a laryngeal or upper tracheal foreign body and the child may be obtunded. In this rare situation the foreign body can be removed with a laryngoscope without anesthesia [7].

Careful preoperative analysis and planning are important components of a successful procedure. The treatment of choice for foreign bodies of the airway is prompt rigid bronchoscopic removal. Rigid open tube bronchoscopes provide two distinct advantages over flexible fiberoptic endoscopes. They allow for control of the airway and provide control of the foreign body. Hopkins rod-lens telescopes can be placed through the bronchoscope to obtain a close up view of the foreign body. Frequently, optical forceps can be used to remove the foreign body. These optical forceps systems are used with the Hopkins rod-lens telescope.

Rarely there are indications for open surgical removal of difficult airway foreign bodies via tracheostomy or thoracotomy [10]. Complications of aspirated foreign bodies can include atelectasis, pneumonia, lung abscess, pneumothorax, pulmonary edema, bronchial hemorrhage and airway obstruction.

### CASE PRESENTATION

A previously healthy 3 year-old female was found near a bowl of peanuts at a Christmas party. She was coughing and wheezing and in a moderate amount of distress. She was taken to the emergency room for evaluation. She was found to have wheezing in the right chest on auscultation. Chest radiograph was normal.

Based on the history and physical exam, the patient was taken to the operating room and underwent rigid bronchoscopy. A peanut was visualized in the right mainstem bronchus (Fig. 1) and removed using optical forceps. After removal of the foreign body, significant mucosal irritation and superficial ulceration were noted (Fig. 2). A second foreign body was found in a distal right segmental bronchus (Fig. 3). This was removed

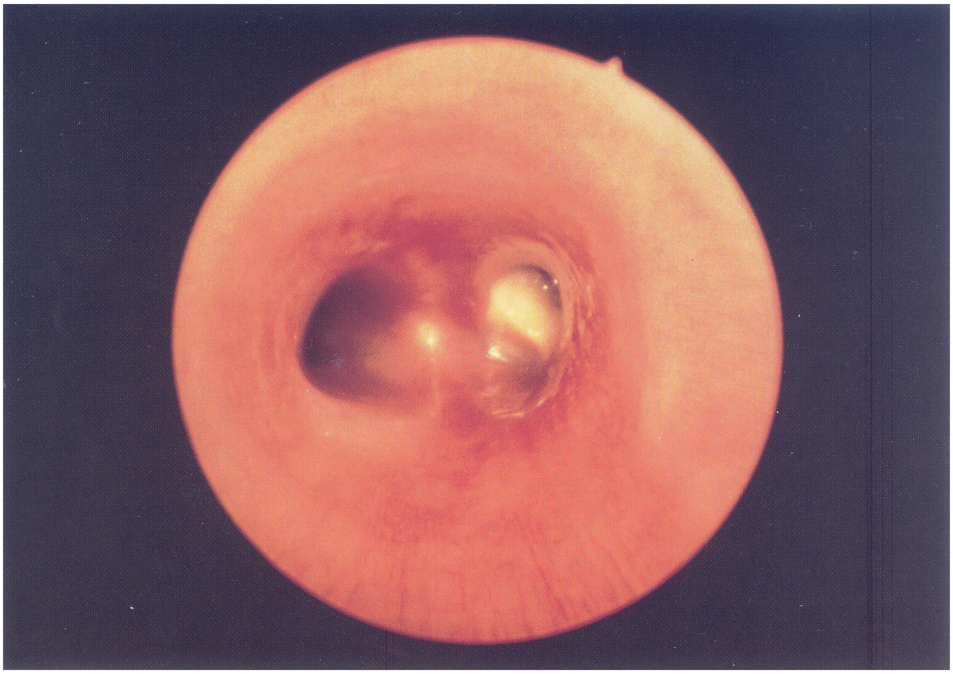

FIGURE 1 A peanut was visualized in the right mainstem bronchus and removed using optical forceps.

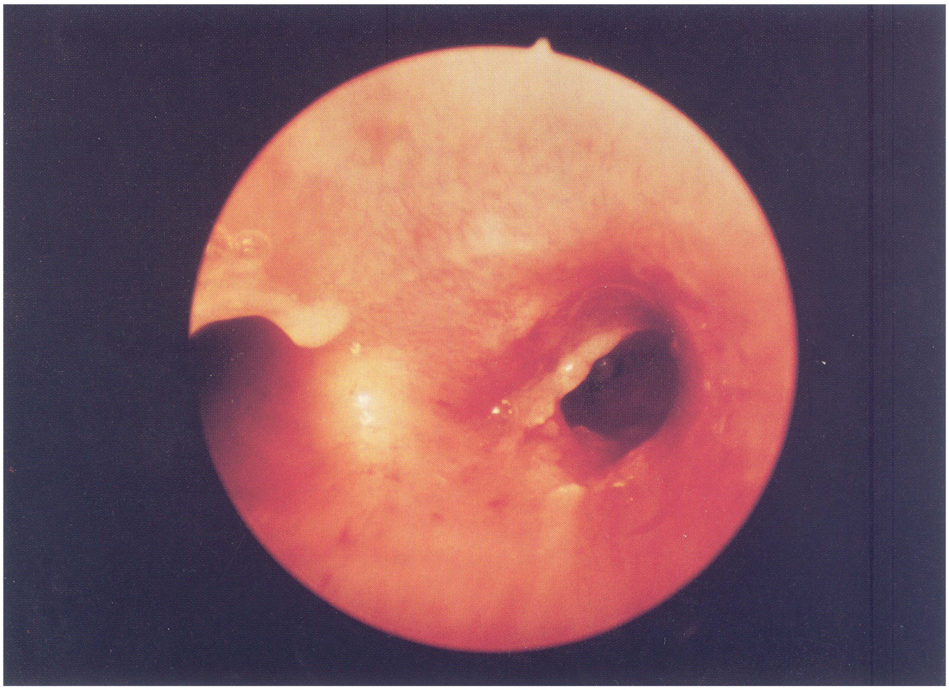

FIGURE 2 After removal of the foreign body, significant mucosal irritation and superficial ulceration were noted. A thick exudate can also be seen. In the distance a second peanut fragment can be seen.

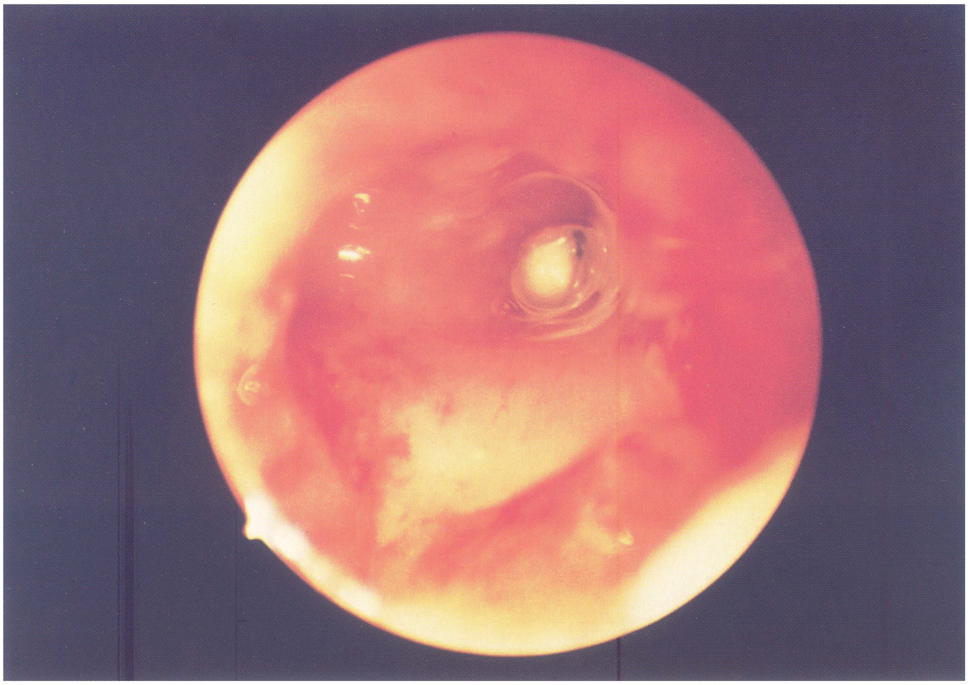

FIGURE 3 A second foreign body is seen in the distal right segmental bronchus. This was removed with optical forceps.

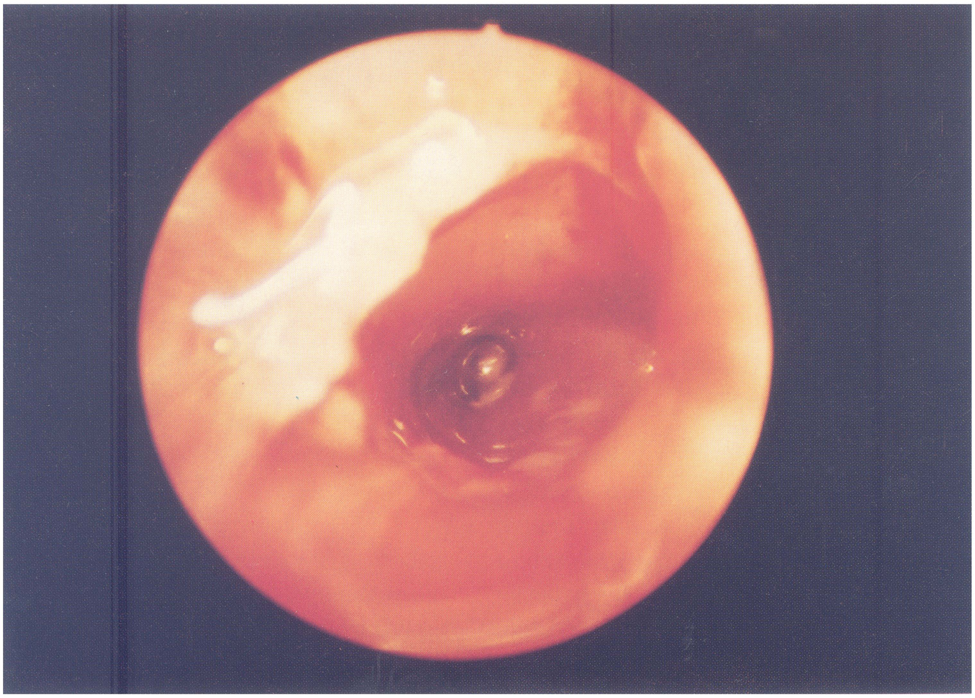

FIGURE 4 The mucosa had the same erythema and edema as demonstrated in Fig. 2.

with optical forceps and the mucosa had the same redness and edema (Fig. 4).

There were no complications and the child was transported in stable condition to the recovery room. After recovery the child was taken to the ward and discharged to home the following day.

### DISCUSSION

This case demonstrates several important points. Hughes *et al.* found that 9% of patients with airway foreign bodies had multiple foreign bodies [9]. It is important to perform a second look after the initial foreign body has been identified and safely removed. A missed second foreign body can result in complications such as lung abscess and may require additional operative procedures at a later setting. The second look also gives the endoscopist a chance to assess airway patency and mucosal reaction at the site where the foreign body was removed.

Figures 2 and 4 demonstrate the intense irritation and edema caused by peanuts. The mucosa is edematous and erythematous with a thick exudate. The oils in nuts and vegetable matter are potent mucosal irritants which may predispose patients with these foreign bodies to pulmonary problems postoperatively [8]. Nuts, beans, and seeds can also greatly enlarge in size when they absorb water. Then they may become soft and fragment easily. These factors can make managing foreign bodies of organic matter more challenging to the endoscopist. Aggressive pulmonary care and antibiotics in these patients may help to prevent pneumonia or to shorten its course [8].

The Hopkins rod-lens telescopes have greatly improved the diagnosis and removal of airway foreign bodies. Specifically, optical forceps with mounted Hopkins rod-lens telescopes have made the removal of some airway foreign bodies safer. These new technologies have probably contributed to decreased complication rates associated with bronchial foreign body removal [4]. This new equipment has also resulted in fewer missed or incomplete bronchial foreign body removals [4]. However, optical forceps are often inadequate for removing foreign bodies from distal small segmental bronchi or for removing foreign bodies from the smaller airways of infants.

### CONCLUSIONS

The removal of the vast majority of airway foreign bodies is performed through the open rigid bronchoscope. New equipment such as the Hopkins rod-lens telescope and optical forceps have improved visualization and made the removal of airway foreign bodies easier in certain instances. The endoscopist needs to be aware of the possibility of a second airway foreign body and perform a second look after the original foreign body is successfully removed. Vegetable matter can swell; it can cause an inflammatory reaction of the mucosa and can easily fragment making its removal more difficult.

### *References*

[1] Killian, G. Direct endoscopy of the upper air passages and oesophagus; its diagnostic and therapeutic value in the search for and removal of foreign bodies. *J. Laryngol. Rhinol. Otol.* 1902; **17**: 461.

[2] Jackson, C. Foreign bodies in the trachea, bronchi and oesophagus – the aid of oesophagoscopy, bronchoscopy, and magnetism in their extraction. *Laryngoscope* 1905; **15**: 257.

[3] Jackson, C. and Jackson, C.L. *Diseases of the Air and Food Passage of Foreign Body Origin*. Philadelphia, WB Saunders, 1936.

[4] Inglis, A.F. and Wagner, D.V. Lower complication rates associated with bronchial foreign bodies over the last 20 years. *Ann. Otol. Rhinol. Laryngol.* 1992; **101**: 61–66.

[5] Mofenson, H.C. and Greensher, J. Management of the choking child. *Pediatr. Clin. North Am.* 1985; **32**: 183–192.

[6] Standards and Guidelines for cardiopulmonary resuscitation (CPR) and emergency cardiac care (ECC). *JAMA* 1986; **255**(21): 2905–89.

[7] Bluestone, C.D., Stool, S.E. and Kenna, M.A. *Pediatric Otolaryngology*. Philadelphia, WB Saunders, 1996.

[8] McGuirt, W.F. *et al.* Tracheobronchial foreign bodies. *Laryngoscope* 1988; **98**: 615–618.

[9] Hughes, C.A., Baroody, F.M. and Marsh, B.R. Pediatric tracheobronchial foreign bodies: historical review from the Johns Hopkins Hospital. *Ann. Otol. Rhinol. Laryngol.* 1996; **105**: 555–561.

[10] Marks, S.C., Marsh, B.R. and Dudgeon, D.L. Indications for open surgical removal of airway foreign bodies. *Ann. Otol. Rhinol. Laryngol.* 1993; **102**: 690–694.

